# Perianal Tuberculosis: A Case Report and a Review of the Literature

**DOI:** 10.1155/2012/852763

**Published:** 2012-12-30

**Authors:** K. Ibn Majdoub Hassani, S. Ait Laalim, I. Toughrai, K. Mazaz

**Affiliations:** Department of Surgery, University Hospital Hassan II, Faculty of Medicine and Pharmacy of Fez, Sidi Mohamed Ben Abdellah University, Route de Sidi Hrazem, Fez 30000, Morocco

## Abstract

Extra pulmonary tuberculosis accounts for less than 15% of all cases of tuberculosis whereas the Intestinal one constitutes less than 1% of the extrapulmonary forms of the disease. The lesions of abdominal organs are more common while they rarely occur in the anoperineal area for the spread of the disease to the anus is extremely rare. We report a case of a 37-year-old male patient with large bilateral infected perianal tubercular ulcerations as well as pulmonary and peritoneal tuberculosis. The treatment was both surgical and medical and the therapy lasted for seven months. After six months from the beginning of the treatment, the lesion had totally disappeared and there is still no recurrence after one year of followup. Tuberculosis should generally be taken into consideration in the differential diagnosis of the ulcerative lesions of the anal and perianal regions for these lesions do occur in the said areas despite their rarity. The treatment is usually both surgical and medical so as to get excellent results.

## 1. Introduction 

Extra pulmonary tuberculosis or (TB) accounts for less than 15% of all cases of tuberculosis [1, 2], while the intestinal one constitutes less than 1% of extrapulmonary forms of the disease [3]. The lesions of abdominal organs are more common while the anoperineal localization rarely occurs. Symptoms and signs of anal pain or discharge, as well as multiple or recurrent fistula in ano and perineal ulcerations, are not characteristically distinct from other anal lesions especially in Crohn's disease. In addition, tuberculosis of the gastrointestinal tract usually occurs as a result of a spread from tuberculosis foci in the lungs. Ingestion of the bacilli from sputum may lead to invasion of the intestinal wall. Positive diagnosis of anal TB relies on both histological and bacteriological assessments. Polymerase chain reaction (PCR) and culture confirm the diagnosis of TB as well.

## 2. Clinical Observation 

A 37-year-old male patient was admitted with a history of perianal discharge and ulceration for the last four months. According to his medical history, he was treated for a perianal abscess which was incised and drained one year ago, yet, despite the initial healing, it recurred two months later.

During the clinical examination at the admission, the patient, with a weight of 40 kg was relatively dehydrated and had tachycardia (pulse rate, 110/min). Physical examination revealed body temperature 38,5°C, and arterial pressure of 09/06 mmhg. No lymphadenopathy was found on palpation; in addition abdominal examination revealed a generalized tenderness. 

**Figure 1:**
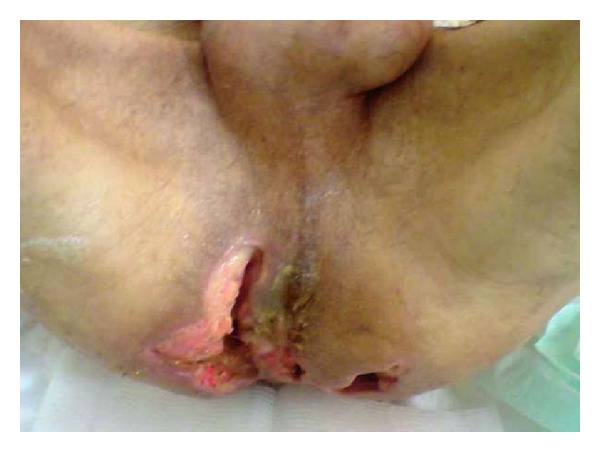
Perianal region examination showing large bilateral infected ulcerations followed by pus.

The perianal region examination showed large bilateral infected ulcerations followed by pus (Figure 1). The digital rectal one revealed no pathological findings except a slight sphincter hypotonia. Anoscopy was normal and no fistulas were noted. The rectosigmoidoscopy showed no abnormalities as well. 

Laboratory investigations showed a hemoglobin level of 09.3 g/dL, a raised white blood cell count of 12,300/*μ*L in addition to high C-reactive protein and elevated erythrocyte sedimentation rate of 50 mm/h. The other laboratory data were within normal limits.

**Figure 2:**
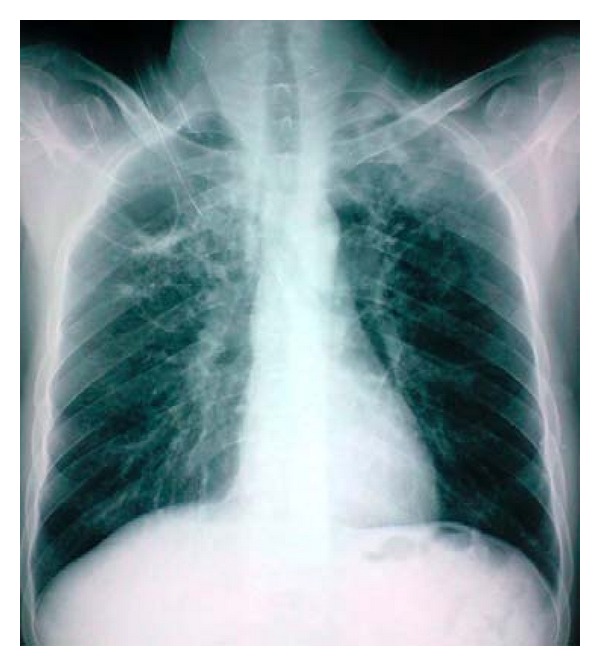
Thoracic X-ray revealed bilateral nonhomogenous infiltrations and cavitary lesions in the right lung.

**Figure 3:**
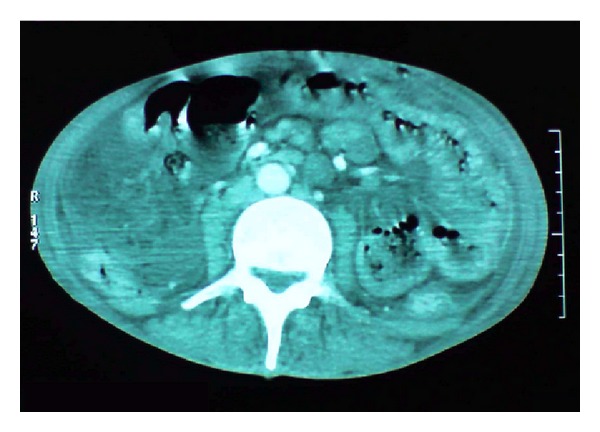
The contrast-enhanced computed tomography scan of the abdominal and pelvic region showed multiple mesenteric and retroperitoneal necrotic adenopathies.

The abdominal X-ray revealed bilateral nonhomogenous infiltrations and cavitary lesions in the right lung (Figure 2). A contrast-enhanced computed tomography scan of the abdominal and pelvic region showed multiple mesenteric and retroperitoneal necrotic adenopathies (Figure 3). The patient was taken to the operating room where a diagnostic laparoscopy was performed and thus showed a thickened and hyperemic peritoneum with Multiple and yellowish white granulations diffusely distributed on the parietal peritoneum and the omentum. Multiple mesenteric adenopathies were noted too. Multiple biopsies of the peritoneum granulations and a diverting sigmoidostomy were performed. 

**Figure 4:**
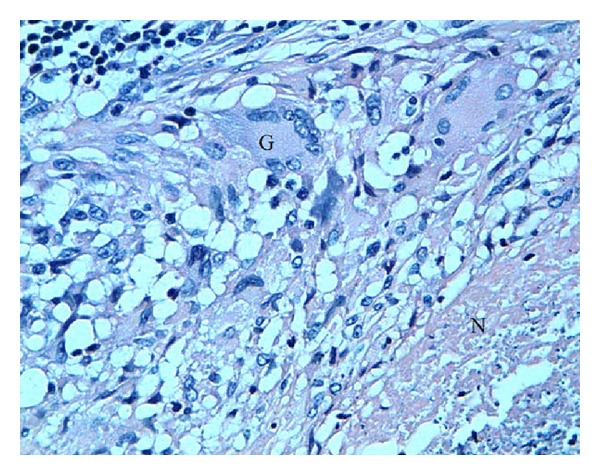
HES × 300, epithelioid granulomas multinucleated giant cells (G), with caseous necrosis (N).

The perianal lesion was cured and biopsy material was taken. Histological examination of all biopsies showed epithelioid granulomas and Langhans' type multinucleated giant cells, with the presence of caseous necrosis (Figure 4). Culture swabs from the perianal lesion confirmed mycobacterium tuberculosis. Examination of sputum samples for tuberculosis was performed but no *Mycobacterium tuberculosis* was found. A tuberculosis skin (PPD test) revealed a positive reaction with 22 mm of induration. The human immunodeficiency virus antibody test was negative.

**Figure 5:**
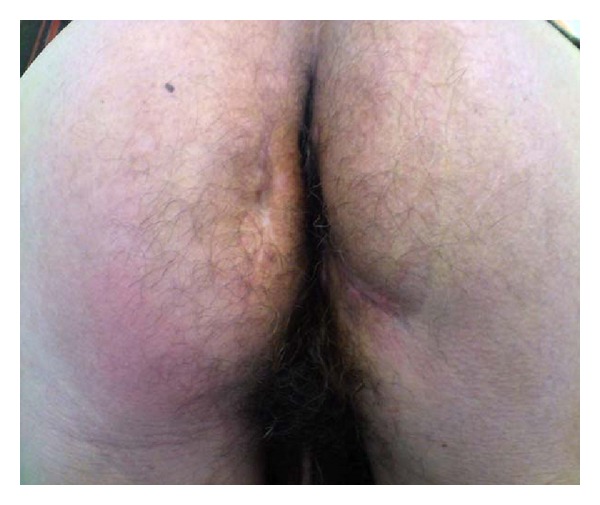
Perianal region examination after six months from beginning of treatement showing complete disappearing of the ulcerations with only a bilateral mild granular region.

We made a diagnosis of perianal tuberculosis with pulmonary and peritoneal involvement and started antituberculous treatment that is consisting of isoniazid, rifampicin, pyrazinamide, and ethambutol for the first two months. No side effects occurred. The patient symptoms vanished and the perianal ulcer began to heal within the first month of treatment. Therapy with isoniazid and rifampicin took seven months. After six months of treatment, the lesion had disappeared, and only a bilateral mild granular region remained (Figure 5). Closure of the protective sigmoidostomy was performed. Now the patient is healthy and having no symptoms. Luckily, there is still no recurrence after one year of followup.

## 3. Discussion

Tuberculosis of the gastrointestinal tract constitutes for 1% of all cases of tuberculosis. It may involve any part of the gastrointestinal system, such as the peritoneum, stomach, duodenum, ileocaecal region, colon, rectum, and anus. Yet, tuberculous peritonitis is the most common. Reference [1, 2] in addition, the most frequently involved site of intestinal tract is the ileocaecal region (85%) [3].

Involvement of the appendix and jejunum is uncommon, and the spread to the anus is much rarer [4]. The anoperineal region is rarely involved in tuberculosis and constitutes less than 1% of all intestinal involvements [5].

Anoperineal TB is commonly seen in men (4/1 ratio) and usually starting from the 4th decade of one's life. It occurs secondary to or coexisting with a pulmonary lesion [5–7] which may be revealed later [8] or may not. Horland and Varkey presented two cases diagnosed as anal tuberculosis associated with pulmonary tuberculosis [5]. Sultan et al. documented data of seven cases of anoperineal tuberculosis observed between 1982 and 1999. An association with pulmonary tuberculosis was found in each case, as well [9].

However, pulmonary tuberculosis may not be present. Intestinal and anoperineal disease may develop by reactivation of the latent focus [2].

Anal contamination generally develops with the ingested airway secretions containing high amounts of *Mycobacterium tuberculosis* bacilli. The other postulated mechanisms by which tubercle bacilli reach the perianal region are hematogenous spread from the primary lung focus in childhood with later reactivation and a direct spread from the adjacent organs, through lymph channels from infected nodes [3, 10]. Sometimes, this comes as a result of inoculation and ingestion of contaminated food [5, 11].

There are five types of anal and per anal involvement of tuberculosis. They can be revealed as ulcerative, verrucous, lupoid, military, and fissure forms [12]. The most common type of anal TB is the ulcerated form which is typically presented as a superficial ulceration that is not hardened. And that tends to have well-defined boundaries with a hemorrhagic necrotic base that is granular and covered with thick purulent secretions of mucous. The lesion may either be very painful or the patient may have few symptoms [13]. The lesion of our patient was ulcerative. The verrucous type tends to extend into the anal passage from the perianal region with a development pattern similar to that of a wart. However, it may appear as a haemorrhoidal nodule, perianal abscess or anal fistula [14, 15].

Perianal cutaneous ulcerations in tropical countries are due to multiple causes: bacterial, viral, and parasitic ones. the differential diagnosis of the tuberculosis ulcerative lesions in the perianal region are Crohn's disease, anorectal abscesses associated with mixed flora, amoebiasis, sarcoidosis, syphilis, lymphogranuloma venereum, malignancies, and foreign body reactions [3, 16].

Few other clinical conditions mimicking tuberculosis are hidradenitis suppurativa, bartholinitis, radiation injuries, lymphomas, and antibiomass. The necessity of a careful study in differentiating the lesion from carcinoma could not be overemphasized [10].

 Differentiating between perianal tuberculosis and Crohn's disease may be difficult. For both conditions have certain similar features including colonic skip lesions, ileocaecal spread and granulomas on histological examination. These two diseases may be difficult to distinguish from each other by macroscopic evaluation. That is why, microscopic examination is needed. When tuberculosis is considered, a biopsy needs to be taken from the lesion; acid fast staining and polymerase chain reaction should be used for a rapid and accurate diagnosis. Finally, cultures are needed to confirm the diagnosis and susceptibility testing [2].

The diagnosis of anal TB is difficult [17] and may be unexpected [18]. The tuberculin skin test remains a valuable guide because it is positive in 75 percent of cases, a negative tuberculin skin test in a patient who is not immunodepressed that is associated with a normal lung X-ray makes the diagnosis of anal TB improbable [14]. Positive diagnosis depends on histologic or bacteriologic analysis. The histological lesion usually involves the epithelioid and giant cell tubercles around the zone of caseous necrosis, with the pathognomic feature being caseating the thing that is not constant. Differentiating the condition from Crohn's disease with anoperineal localization can be difficult if there is no caseation or direct evidence of acid fast bacilli [19]. Bacteriological analysis is done by identification of Koch's bacilli by direct examination (Ziehle-Neelsen stain) and culture [9]. The diagnosis is supported by the clinical response to an antitubercular therapy [20]. The detection of mycobacterial DNA in clinical samples by polymerase chain reaction (PCR) is a promising approach for the rapid diagnosis of tuberculous infection [21, 22], which can detect the presence of bacterial DNA in 48 h with high sensitivity and specificity when testing several samples [23].

The treatment of the anal tuberculosis is medical. Surgical procedures are needed if there is a fistula or abscess [9]. The ulcerative lesions of the anus associated with tuberculosis regress in few weeks following the treatment.

## 4. Conclusion

Tuberculosis lesion in and around the anus is very rare, but common at the same time. The latter may manifest as an ulcerative, verrucous, lupoid, and miliary form. The anoperineal involvement may be associated with pulmonary and abdominal tuberculosis either as an extension of the original lesion or due to its spread via the lymphatics. Primary lesions are rare but existing. That is why tuberculosis should generally be taken into consideration in the differential diagnosis of the ulcerative lesions of the anal and perianal regions. The treatment is twofold: surgical for the suppuration and medical for the tuberculosis with excellent results.
